# Diagnosis and Treatment of Pulmonary Coccidioidomycosis and Paracoccidioidomycosis

**DOI:** 10.3390/jof9020218

**Published:** 2023-02-07

**Authors:** Paula Massaroni Peçanha-Pietrobom, Andrés Tirado-Sánchez, Sarah Santos Gonçalves, Alexandro Bonifaz, Arnaldo Lopes Colombo

**Affiliations:** 1 Department of Medicine, Division of Infectious Diseases, Federal University of São Paulo (UNIFESP), São Paulo 04039032, Brazil; 2 Dermatology Service & Mycology Department, Hospital General de México, “Dr. Eduardo Liceaga”, Mexico City 06726, Mexico; 3 Internal Medicine Department, Hospital General de Zona 29, Instituto Mexicano del Seguro Social, Mexico City 07950, Mexico; 4 Department of Pathology, Infectious Diseases Postgraduate Program, Federal University of Espírito Santo (UFES), Vitoria 29043900, Brazil

**Keywords:** paracoccidioidomycosis, coccidioidomycosis, endemic mycoses, antifungal treatment

## Abstract

Coccidioidomycosis (CM) and paracoccidioidomycosis (PCM) are systemic mycoses that are highly endemic in Latin America and have recently been included on the World Health Organization (WHO) Fungal Priority Pathogens List. *Coccidioides immitis* and *Coccidioides posadasii* are recognized as etiological agents of CM, with peculiarities in their geographic distribution. The genus *Paracoccidioides* now includes *Paracoccidioides lutzii* and the *Paracoccidioides brasiliensis* complex, which encompasses four phylogenetic species. In both diseases, pulmonary signs and symptoms are the main reasons for patients to seek medical assistance, and they are frequently misdiagnosed as tuberculosis. In this paper, we present a critical view of the strategies for diagnosis and clinical management of CM and PCM. Over the past few decades, there has been an increase in the number of reports of endemic fungal infections in areas previously thought to be “non-endemic” due to climate change and increased travel, among other factors. Learning to recognize their main epidemiological aspects and clinical manifestations is crucial so that clinicians can include them in the differential diagnosis of lung disease and avoid late diagnosis.

## 1. Introduction

Endemic mycoses include fungal infections with circumscribed geographic distribution, as their etiological agents require specific climatic and environmental conditions for survival and reproduction. Etiological agents are thermally dimorphic fungi capable of causing diseases in healthy and immunocompromised hosts [1,2].

Coccidioidomycosis (CM) and paracoccidioidomycosis (PCM) are two highly endemic systemic fungal infections in America. Accurate incidence rates of CM and PCM remain unclear as most endemic countries do not demand compulsory notification by medical centers [2]. Over the past few decades, there have been increasing reports of infections caused by endemic fungi in areas previously thought to be “non-endemic”. This phenomenon, which has also been documented with PCM and CM, is multifactorial, and potential reasons for it include climate change, increased ability to recognize the fungal disease, an ever-increasing number of patients with comorbidities associated with depressed T cell immunity, and global factors such as migration and increased travel [2,3].

The taxonomy of fungal agents has undergone notable changes with the incorporation of molecular studies, thereby providing a more accurate and reliable classification. In this scenario, *Coccidioides immitis* and *Coccidioides posadasii* are recognized as etiological agents of CM, with peculiarities in their geographic distribution [4]. The genus *Paracoccidioides* now includes *Paracoccidioides lutzii* and the *Paracoccidioides brasiliensis* complex, which encompasses four phylogenetic species, including *Paracoccidioides brasiliensis sensu stricto* (S1a and S1b), *Paracoccidioides americana* (PS2), *Paracoccidioides restrepiensis* (PS3), and *Paracoccidioides venezuelensis* (PS4) [5,6,7,8].

CM and PCM are acquired by the inhalation of fungal spores through the respiratory tract, and pulmonary symptoms account for the main clinical presentation of both endemic diseases [3]. Consequently, they can be initially misleading and confused with community-acquired pneumonia and other diseases, such as pulmonary tuberculosis (TB), making it difficult to distinguish them clinically and radiologically [3,6,8,9,10]. A recent study assessing 213 smear-negative patients with clinical suspicion of TB assisted in Brazilian Amazon medical centers found that fungi (mainly *Aspergillus* spp. and *Paracoccidioides* spp.) were the etiologic agents in 7% of cases [11]. Another retrospective study investigating the history of clinical management of 227 adult patients with PCM (chronic form) found that 16% of them had been previously treated for TB. Of note, in the same cohort, the authors documented the concomitance of PCM and TB in 8% of cases [12]. The coexistence of TB with either CM or PCM has been extensively reported by different investigators. Misdiagnosis of pulmonary mycosis as TB in Latin America incorrectly increases the official number of cases attributed to mycobacteriosis, results in delayed treatment of fungal diseases, increases morbidity, and is associated with economic loss [13,14].

Recently, CM and PCM were included on the World Health Organization (WHO) Fungal Priority Pathogens List due to their endemicity in the Americas, high morbidity, and potential for sequelae that negatively impact the quality of life [15]. Regarding PCM, there have been some efforts to include this fungal infection in the WHO list of neglected diseases, as most affected patients are poor rural workers with limited access to diagnosis and treatment [13,14,15].

## 2. Coccidioidomycosis

### 2.1. Ecoepidemiology

CM is caused by two cryptic fungal species, *Coccidioides immitis* and *Coccidioides posadasii*. The first may be found in southern California and the second in other regions, primarily in Arizona, the Western United States, and the Americas (Mexico, Central, and South America). CM involves chronic pulmonary disease with extrapulmonary (cutaneous and visceral) manifestations in high-risk patients. It is also called valley fever, which refers to the San Joaquín Valley in California, US, and is endemic [16,17,18,19,20].

All the endemic areas of CM in the Americas are semi-desert areas, with clay, sandy, and alkaline soils, rainfall ranging from 150 to 500 mm per year, and an average temperature of 28 °C. The fungus is usually located 5–30 cm underground in xerophytic plants (cacti), shrubs, and plants such as Larrea tridentata (“gobernadora”), and can also be found in rodents such as mice, opossums (*Perognathus*), and some reptiles. Clusters of CM have been associated with the meteorological phenomenon “El Niño”. Intense rainy seasons facilitate fungal growth in endemic areas, and spores are easily dispersed when soils are dry [16,18,19,21,22].

**Figure 1:**
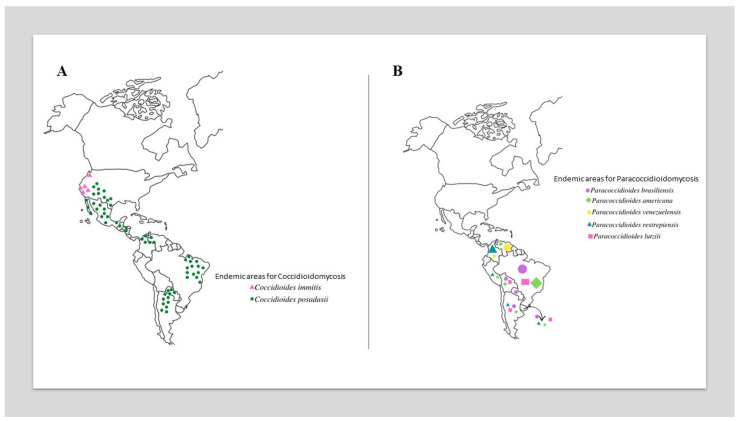
Global geographic distribution of *Coccidioides* species (**A**) and *Paracoccidioides* species (**B**). In Figure 1B, the size of the elements is proportional to the prevalence of species in different countries.

The most critical epidemiological zones for CM include the southwestern US (California, Arizona (with 60% of reported infections), Utah (Mojave Desert), Texas, New Mexico, and Nevada) [16,17,21,22,23,24,25,26,27] and northwestern Mexico (Baja California Norte, Sonora (Sonora Desert), Sinaloa, Nuevo León, Chihuahua, and Coahuila) [19,28,29]. In Central America, small foci have been reported in Guatemala, El Salvador, Nicaragua and Honduras. In South America, endemic foci are present in the Guajira Peninsula, which is shared by Colombia and Venezuela, a growing focus is on northeastern Brazil (Ceará, Piauí, Maranhão, and Bahia), and the focus of the first report is in northern Argentina (Jujuy, Río Negro, Catamarca, and Valle Central) (Figure 1A) [29,30,31,32]. Traveling to defined endemic areas (e.g., ecotourism) increases the risk of acquiring infection [32].

The main entry route for infection is inhalation, but CM may also rarely be acquired through skin implantation (<10%) [16,17,19]. The infection occurs at any age, but it is more common among young adults, mostly male patients (male: female ratio, 4:1), with apparently no racial predilection. Other evidence suggests that disease manifestations are more frequently found among African Americans and Asians, and Latino heritage is reported as a risk factor. This is probably due to the expression of haplotypes such as HLA class II DRB*1301. The incubation period of the disease ranges from days to months [4,21,23,33].

The Centers for Disease Control (CDC) has reported an increase in the incidence of cases of CM as well as an expansion of its geographic range, probably due to climate change and a significant number of populations at risk in endemic areas. Between 1995 and 2000, the incidence of CM was 2.4 cases per 100,000 inhabitants; however, between 2000 and 2006, the incidence tripled to 8.0 cases per 100,000 inhabitants. In particular regions, such as the San Joaquín Valley and Kern County, hyperendemicity may reach 53.9 to 150 cases per 100,000 inhabitants, respectively [20,23,27,34]. In Mexico, endemicity is similarly high, with an estimated incidence of 0.5 to 2.6 cases per 100,000 inhabitants and 500 to 1500 new cases per year [29]. In Brazil, endemicity is lower than that in North America, and a total of 800 cases have been reported, mainly in areas of the state of Ceará [29,32]. Notably, CM is a non-notifiable disease in most Latin countries, and its actual incidence is unknown [28].

### 2.2. Pathogenesis

*Coccidioides immitis* and *C. posadasii* cause phenotypically similar diseases but exhibit genetic peculiarities and different geographic distributions, despite some overlaps that may occur [17,19,22]. Biological differences between both agents are probably secondary to their adaptation to different niches and habits in nature [35]. The life cycle of *Coccidioides* spp. begins with filamentous phase reproduction and formation of mature arthroconidia (infective phase), which are easily detached from the mycelia, released, and aerosolized with further dissemination in the environment. Once infected propagules are inhaled by humans, they can penetrate the lungs and deposit in a terminal bronchiole and alveoli where they undergo enlargement, developing 10–20 µm spherical structures which grow until they double in size due to nuclear duplication phenomena. Endospores (1–4 µm) develop internally, forming mature spherules (50–70 µm). This maturation phenomenon induces the weakening of the fungal membrane, allowing the ejection of endospores into the host tissues and fluids. Fungal infection is contained when the host organizes an efficient cellular immune response (granuloma with macrophages, giant cells, and lymphocytes), preventing further multiplication and dissemination of fungal elements. However, latent foci containing viable fungal elements may persist for long periods, explaining the late reactivation of the disease, as described in other pulmonary endemic mycoses and TB [19,36,37,38,39,40,41].

### 2.3. Clinical Aspects

**Figure 2:**
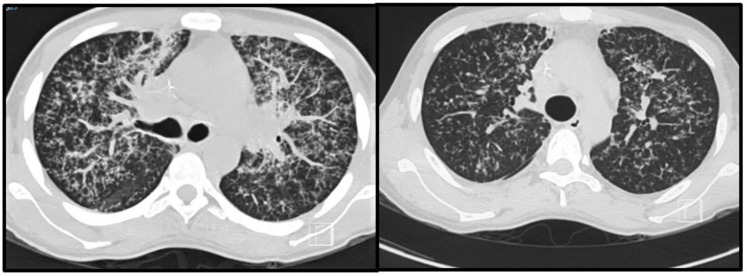
Pulmonary images of a 19-year-old male patient with subacute pulmonary coccidioidomycosis: axial thoracic CT showing bilateral peribronchovascular and perilymphatic nodules in both lungs.

Most patients will develop an asymptomatic infection (60% to 75%) that is only detectable by intradermal reaction (coccidioidin). The primary and most common clinical manifestation of CM is an acute pulmonary illness resulting from inhalation of arthroconidia. Acute symptomatic forms of the disease usually present 3 weeks after inoculation and are constituted by mild flu-like respiratory symptoms, with moderate fever, headache, chills, night diaphoresis, and dry cough [4,16,20,38,39,40,41]. Because respiratory manifestations are indistinguishable from bacterial pneumonia, the diagnosis of pulmonary CM is often delayed for at least 1 month, prolonging unnecessary exposure to antibiotics. Radiologically, small pulmonary nodules, rare cavitary lesions, pneumonic infiltrates, and pleural effusions have been observed. In patients with multiple risk factors, symptoms are severe and include constant fever, cough with mucopurulent expectoration, and frank hemoptysis. Some patients develop arthralgia (“desert rheumatism”) [16,17,38,39]. Hypersensitivity reactions, such as erythema nodosum (lower limbs), erythema multiforme, and lymphadenopathy, may occur during febrile illness and are more frequent in women [19]. Figure 2 illustrates a patient with subacute pulmonary CM exhibiting multiple peribronchovascular and perilymphatic nodules in both lungs.

The progressive form of CM with extrapulmonary manifestations is documented in less than 2% of patients and requires the coexistence of underlying conditions associated with T-cell depression. Dissemination of CM may be a consequence of exogenous reinfection or dissemination of the primary form. However, acute and chronic pulmonary symptoms are the most common reasons that patients seek medical help [10,42]. Patients often develop fever, chest pain, productive cough with abundant exudates, purulence, and hemoptysis. Radiologically, pulmonary infiltrates and adenopathy (hilar and mediastinal in severe cases) are observed; exceptionally, cavities may also form. The most serious pulmonary manifestation is the miliary type, which is indistinguishable from miliary TB [4,17,18,38]. Extrapulmonary CM can be primarily cutaneous due to implantation or secondary to pulmonary illness, and meningeal manifestations may rarely occur, especially in high-risk patients with depressed T-cell immunity [10,19,43,44,45].

During the coronavirus SARS-CoV-2 pandemic, we learned that severe COVID-19 might be added to the long list of other risk conditions that can complicate the natural history of CM, including diabetes, smoking, patient ethnicity (Hispanics and Afro-Americans), immunosuppression, and pregnancy [23,46]. Moreover, SARS-CoV-2-related immune dysregulation may reactivate latent CM [47].

### 2.4. Laboratory Diagnosis

**Figure 3:**
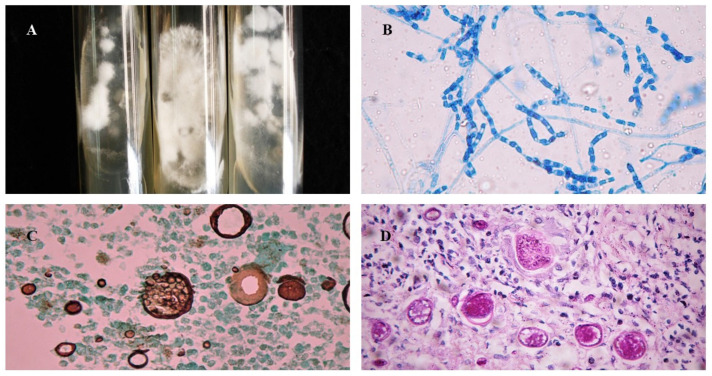
Morphological aspects of *Coccidioides* spp.: (**A**) Colonies of filamentous fungi in Sabouraud Dextrose Agar cultivated at 25 °C after 2 weeks; (**B**) Arthroconidia and septate hyaline hyphae of *Coccidioides* at 25 °C, stained with cotton blue, 40×; (**C**) Histopathological examination of biopsy stained with Grocott–Gomori methenamine-silver showing *Coccidioides* spp. spherules with multiple endospores, 40×; (**D**) Histopathological examination of biopsy stained with Periodic Acid Schiff showing *Coccidioides* spp. spherules, 40×.

Mycological diagnosis may be provided by cytology from sputum samples, bronchoalveolar lavage fluid, or, in cases of extrapulmonary involvement, characterization of fungal elements in exudates. The pathognomonic parasitic forms or spherules confirm the diagnosis of CM (Figure 3), but sensitivity depends on specimen quality and observer expertise (see Table 1). Cytopathology is useful, especially when the sample comes from bronchoalveolar lavage or fine-needle aspiration of lung lesions. In general, Papanicolaou staining allows for visualization of the spherules [48,49]. Cultures are also considered the gold standard method for diagnosis but should only be performed in biosafety level three laboratories (Figure 3A). *Coccidioides* spp. usually grow between 4 and 7 days at a temperature of 25 °C to 28 °C in Sabouraud dextrose agar medium, with or without antibiotics. Hyaline hyphae septa with rexolytic arthroconidia are observed under microscopy (Figure 3B) but are often misdiagnosed as *Geotrichum* sp. or *Magnusiomyces* (formerly *Saprochaete* sp.) [4,16,17,50,51,52].

Polymerase chain reaction (PCR) is a convenient test with low time consumption, primarily used in real-time platforms (4–5 h), but is not available in most routine laboratories [53,54]. The performance of PCR-based methods may vary between different biological materials (sputum and cerebrospinal fluid) and “in-house protocols”. PCR assays usually exhibit better performance than cultures [51,52].

Pulmonary and cutaneous histopathology may provide valuable information with the visualization of suppurative granulomas surrounding typical *Coiccidioides* spherules, These elements are better visualized when using periodic acid Schiff (PAS) or Grocott methenamine silver (GMS) stains (Figure 3C) instead of only hematoxylin-eosin (HE) (Figure 3D) [19,27].

The intradermal reaction to coccidiodin is a useful tool only for determining the first contact and rate of individuals exposed to fungi and mapping reactor zones. A positive skin test may also be used to determine disease prognosis when it is associated with serological results and clinical aspects of the disease [19,27,51]. A significant number of diagnoses of CM in endemic areas are based on serological test results, including IgG (better performance) and IgM-specific antibody levels that are detected by enzyme-linked immunoassays (ELISA) and other platforms. Williams and Chiller [23] reported that IgG sensitivity ranges between 47% and 87%, with a specificity of 90% to 96%, compared with IgM sensitivity, which yields lower results and increased false positives. Another antibody titration technique involves direct immunodiffusion (DI), which can also measure both antibody types; however, it is more time-consuming than ELISA. On the other hand, the complement fixation (CF) assay is less sensitive than ELISA and has high specificity rates. This test can be quantitative (by serum titration) and provides precise data. High CF titers and negative intradermal reactions indicate a poor prognosis. CF serology is also helpful for therapeutic monitoring, since titers ≤1:8 indicate a cure. The drawback of CF is that it is a complex and nonstandardized technique. Lateral flow tests (LFA) are simple to perform; however, they exhibit low sensitivity compared with ELISA and CF [23,27,43,44,45,50,51,52].

**Table 1:** The sensitivity, specificity, advantages, and disadvantages of all diagnostic methods for Paracoccidioidomycosis and Coccidioidomycosis.

Diagnostic Test	Sensitivity	Specificity	Advantageous	Disadvantageous	References
Paracoccidioidomycosis					
Mycological					
Direct Microscopy	48–75%	High	Pathognomonic fungal elements may be rapidly identified (mother cell with multiple buds: “Ship’s wheel” or “Mickey Mouse”). It may be performed in exudates of tegumental lesions, sputum, and aspirates of lymph nodes. Simple, fast, and low-cost.	The sensitivity is dependent on the operator’s experience, the quality of the material, and the protocol for processing. Small yeasts without multiple buds can be mistaken for other fungi.	[55,56]
Histopathology	70->5%	High	Pathognomonic fungal elements may be rapidly identified (mother cell with multiple buds: “Ship’s wheel” or “Mickey Mouse”). Tissue response and burden of infection may be evaluated and help to characterize the severity of the disease. Results may take less than 24 or 48 h.	An invasive procedure is required to obtain a biopsy. The sensitivity is dependent on the operator’s experience. The quality of the biopsy sample, and the protocol for processing. Specific fungal stains should be included to improve the detection of fungal elements.	[56,57]
Culture	24->90%	100%	It may be performed in different biologic samples, with higher sensitivities in bronchoalveolar lavage and tissue biopsies. It provides accurate identification of *Paracoccidioides* spp. at the species level and can possibly check antifungal susceptibility and virulence. Low cost (higher if molecular tests were used to identify species)	Exudates and sputum samples may be contaminated with bacteria which may mitigate the sensitivity of the test (all media cultures should contain antibiotics). *Paracoccidioides* spp. are fastidious microorganisms and culture will take an average of 3 to 4 weeks to grow. There are biosafety concerns with the manipulation of *Paracoccidioides* spp.	[55,56]
Immunological					
Antibody (GP43 and GP70) detection by different assays	80–95%	80–100%	They may provide quantitative in addition to qualitative results. The test may help with diagnosis and the laboratory monitoring of response to therapy. Results may be provided in hours or a few days.	Cross-reactions may occur with other fungal infections. Sensitivity is highly dependent on host immunity, clinical form, and methodology of detection. False-negative results may be provided for *P. lutzii* when using *P. brasiliensis* complex antigen preparation and vice versa. No commercial tests are available.	[58,59]
Specific Antigen detection for PCM	90–96%	96–100%	It is highly recommended for testing immunocompromised patients with (higher sensitivity than antibody detection). It is a non-invasive procedure. Results may be provided in less than 24 or 48 h in reference centers.	Cross-reactions may be obtained with heterologous sera. No commercial tests are available, and assays are not available in routine laboratories.	[60,61,62]
Molecular					
PCR based methods	86–100%		It may detect DNA in samples with a low burden of fungal elements. It facilitates the possibility of species identification. Results may be provided in less than 24 or 48 h.	There is no international standardization of the tests. They are not available for most routine laboratories that are located in endemic regions. There is no commercial kit available. Most tests are designed to identify *P. brasiliensis*. There is a lack of standardized tests to detect *P. lutzii* infection in biological samples.	[63,64]
		Coccidioidomycosis			
Mycological					
Direct Microscopy	15–75	High	Pathognomonic fungal elements may be rapidly identified (Cocci spherules with endospores). Testing is low-cost, rapid and easy, and various biological materials can be used.	The sensitivity depends on the operator’s experience, the quality of the material, and the protocol for processing.	[23]
Cytopathology/Histopathology	23–84%	High	Pathognomonic fungal elements may be rapidly identified (Cocci spherules with endospores). Testing is low-cost, rapid and easy, and various biological materials may be tested. The specificity and sensibility of the test may be improved by the inclusion of specific fungal stains.	The sensitivity is dependent on the operator’s experience, the quality of the biopsy material, and the protocol for processing. A biopsy requires invasive procedures.	[23]
Culture	Close to 50%	High	It may be performed in different biologic samples, with higher sensitivities in broncho-alveolar lavage and tissue biopsies. It provides accurate identification of *Coccidioides* spp. at the species level as well as the possibility of checking for antifungal susceptibility and virulence. Testing is low-cost (higher if molecular tests are used to identify species).	Manipulation requires a level 3 biosafety laboratory. Results may require up to 7 days. Contamination of samples with bacteria may mitigate the sensitivity of the test.	[65,66]
Immunological					
Specific Antibody (IgG or IgM) detection	60–100%	90–96%	They may provide quantitative addition to qualitative results. The test may help with diagnosis and the laboratory monitoring of response to therapy. Results may be provided in hours or a few days.	Cross-reactions may occur with other fungi. Sensitivity is highly dependent on host immunity, clinical form, and methodology of detection. Serological tests are commercially available.	[23,67]
CM Specific Antigen tests	28–70%	90–100%	It is highly recommended for immunocompromised hosts where the production of antibodies may be low. Tests are usually performed on urine and serum samples. Results may be provided in hours or a few days.	Serum samples usually present lower sensitivity compared with urine samples. Cross-reaction with *Histoplasma* spp. and *Blastomyces* spp. may occur. Serological tests are commercially available.	[68,69]
Molecular					
PCR based	>80%	High	They may detect DNA in samples with low fungal burden, exhibiting better performance than culture. There is a possibility of species identification as well as characterization of antifungal susceptibility and virulence.	There is a lack of international standardization. Only in-house tests are available in a limited number of reference medical centers.	[70]

### 2.5. Treatment

**Figure 4:**
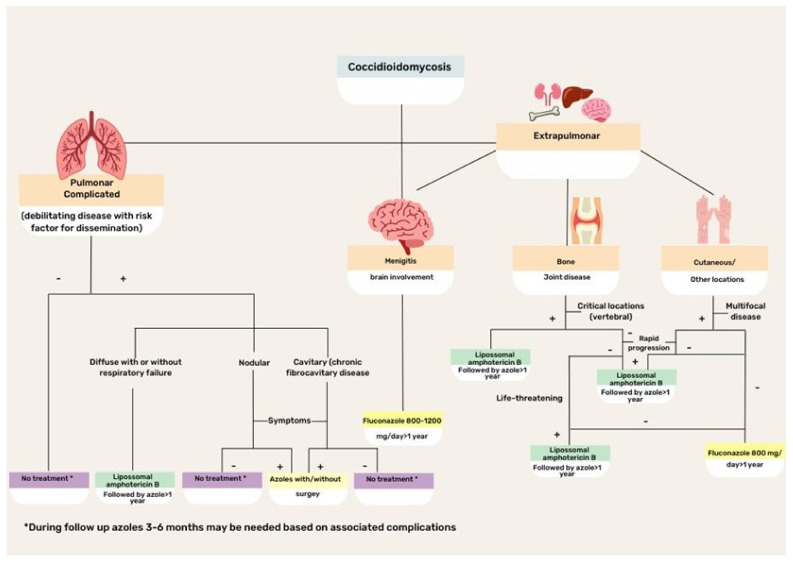
Flowchart illustrating the treatment of different clinical forms of coccidioidomycosis.

CM treatment is based on disease severity and host immunity status (Figure 4). Asymptomatic or mild disease may not require antifungal therapy because the disease is commonly self-limiting in normal hosts. Regarding the specific scenario of HIV coinfection, antifungal therapy is recommended for all patients with HIV infection with clinical evidence of CM and a peripheral blood CD4+ T lymphocyte count <250 cells/µL. Initiation of potent antiretroviral therapy should not be delayed because of concerns regarding coccidioidal immune reconstitution inflammatory syndrome [43].

For many years, amphotericin B (Amph-B) formulation has been the treatment of choice for severe cases of CM. Currently, triazoles are preferred, although Amph-B is used to treat cases that are refractory to triazoles, as well as critically ill patients. Lipid formulations are preferred to the classic deoxycholate form. Recommended doses for liposomal Amph-B are 3–5 mg/kg/day, while for lipid complex Amph-B, 5 mg/kg/day classic or deoxycholate Amph-B is usually prescribed at 0.5–1 mg/kg per day. Side effects, particularly renal and myelotoxicities, can occur with any of the Amph-B formulations, although lipid formulations reduce the risk. Patients with meningitis or brain-affected CM are candidates for intrathecal drug administration [16,18,19,43].

The first treatment option for CM is fluconazole, which is effective and safe for patients with extrapulmonary disease. However, fluconazole-resistant isolates have been increasingly reported and may require therapy with Amph-B formulations. The initial intravenous route of triazole administration is preferred, with a later transition to an oral formulation, with doses ranging between 400 and 800 mg/day. In meningeal cases, fluconazole is effective because of its ability to cross the blood–brain barrier and contain infection [16,23,40,43].

As an alternative, itraconazole might be used, although it is not available in intravenous formulations in most countries in Latin America. The usual dose is 200–400 mg/day, with progressive tapering according to the clinical and mycological responses. Other triazoles that exhibit variable results with CM include voriconazole and posaconazole [19,43,44]. Both options have been used in the salvage treatment of refractory CM with good tolerability and success rates of 65–75% [71].

### 2.6. New Perspectives in the Clinical Management of CM

Nikomicin (nikkomyzin Z) is an antifungal agent that inhibits chitin synthesis. Promising results were observed in a phase II study of dogs with CM. However, well-designed clinical trials are not available to confirm its effectiveness and safety profile in patients with CM [72,73,74,75].

Olorofim is a new antimitotic belonging to the orthomid class that targets the inhibition of pyrimidine synthesis. It has good in vitro activity against *Coccidioides* spp. and a murine model; moreover, it was found to be superior to fluconazole, raising new expectations [76,77].

Considerable efforts have been made to develop vaccines that prevent CM infection in humans and animals. One possibility is to use live attenuated or inactivated microorganisms, but this strategy has led to poor results. The ∆cps1 vaccine provided satisfactory results for veterinary use and has the potential to be explored in human hosts. This vaccine interrupts the maturation of spherules and affects the tissue adaptation of fungi [78,79,80].

## 3. Paracoccidiodomycosis

### 3.1. Ecoepidemiology

PCM is autochthonous in Latin America, with a high incidence, particularly in Argentina, Brazil (accounts for 80%), Colombia, and Venezuela. Cases of PCM have not been reported in Nicaragua, Belize, most Caribbean islands, Guyana, Suriname, or Chile (Figure 1B) [2,81].

For over a century, *Paracoccidioides brasiliensis* has been believed to be the single agent causing PCM. However, molecular advances and phylogenetic analyses have led to the recent classification of the genus *Paracoccidioides*, which currently encompasses the *P. brasiliensis* complex and *P. lutzii*. Most PCM cases are related to infections that are caused by the *P. brasiliensis* complex, which comprises four cryptic species: *Paracoccidioides brasiliensis* *sensu stricto* (S1a and S1b), Paracoccidioides americana (PS2), *Paracoccidioides restrepiensis* (PS3), and *Paracoccidioides venezuelensis* (PS4). There are no substantial differences in their virulence characteristics, clinical manifestations, or antifungal responses to therapy [6,8,82]. *Paracoccidioides brasiliensis* sensu stricto is widespread and the predominant agent of PCM in most South American countries (Figure 1B). *P. lutzii* presents significant antigenic differences from all cryptic species within the *P. brasiliensis* complex, with diagnostic implications, since different antigen preparations are required to detect patients who are infected by both agents [55,83].

Male rural workers aged between 30 and 60 years represent the main population at risk of developing PCM, followed by bricklayers, as both professional activities expose individuals to aerosol-containing soil fungal particles. Living in rural areas or on the periphery of urban centers (overlapping with rural zones) is also a major risk factor for infection [84,85]. The racial predisposition to the development of severe forms of PCM remains controversial. So far, only one study performed in Southern Brazil found a higher prevalence of disseminated lesions in black patients with acute/subacute forms of PCM than in white individuals [86]. Nevertheless, other confounding factors with potential impacts on the natural history of PCM were not accurately considered in their study.

PCM has an estimated occurrence of one to four cases/0.000 inhabitants per year in geographic areas with stabilized endemicity. In contrast to CM, the incidence of PCM does not seem to be increasing, although the lack of compulsory notification of the disease in most Latin American countries precludes precise evaluation of the prevalence and incidence of this fungal disease [81,87,88]. Indeed, there is some evidence suggesting that the hospitalization and mortality rates attributed to PCM are decreasing in reference centers from the Southeast and South Regions of Brazil [89,90]. The mechanization of agriculture, the extensive use of antifungals in agriculture, and the reduction in child labor are all conditions that prevent people from being exposed to the fungus and promote a reduction in the number of new cases in the first recognized areas of PCM high endemicity martine [81,91]. Otherwise, the continuous expansion of rural frontiers, along with the deforestation of native vegetation, has led to a substantial rise in PCM cases in the Central-Western and Northern areas of Brazil, extending to the border with Bolivia. As a result, new hyperendemic areas have been characterized in the state of Rondônia, with an incidence as high as 9.4–40/100,000, and more recently in Tocantins, Pará, and Maranhão [92,93].

In addition, current evidence suggests that several human interventions involving massive land removal may affect PCM epidemiology. This was the case with the substantial rise in PCM infection episodes assessed by skin tests with paracoccidioidin documented after the construction of the Yacyreta hydroelectric plant in northeastern Argentina and an increase in the incidence of acute/subacute PCM cases documented after the construction of the ring road in the Rio de Janeiro metropolitan area in 2016 [94,95].

As with CM, climate change has been implicated in modifying PCM epidemiology. Clusters of acute/subacute cases of PCM were reported after the El Niño phenomenon in 1982/83 in Southeast Brazil and 2009 in northeastern Argentina [96,97].

Outside endemic areas, PCM may present as a traveler’s disease in tourists visiting Latin America. Over 100 PCM cases have been reported in Europe, North America, Asia, and Africa. PCM diagnosis among travelers or immigrants from endemic countries is challenging once the disease develops decades after exposure [98,99,100,101].

PCM is mostly documented in immunocompetent patients, but its natural history may be negatively affected by T-cell immunosuppression. Although rare, most cases of PCM in immunocompromised patients have been documented as coinfections with HIV, followed by oncologic patients and solid organ transplant recipients [102,103,104,105,106,107,108,109].

### 3.2. Pathogenesis

**Figure 5:**
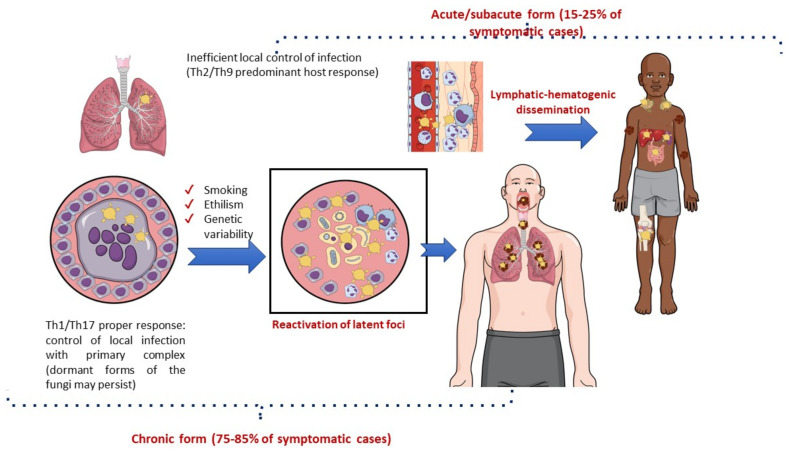
Natural history of paracoccidioidomycosis with a focus on pathogenesis.

The immunological pattern of the host response to infection is a major determinant of the clinical manifestations of PCM [59,110]. The lungs are the usual portal of entry for *Paracoccidioides* spp., where an efficient Th1 cellular response with a granulomatous reaction prevents disease development and fungal dissemination. Most cases of infection are asymptomatic (>98%) or manifest clinically with nonspecific symptoms, leaving behind scar focal lesions that may contain latent viable yeast cells [107,108]. Symptomatic patients may develop acute/subacute manifestations (a juvenile form of PCM) or chronic diseases (an adult form of PCM). The lung manifestations of PCM are generally restricted to the chronic form of the disease (Figure 5) [86,111].

The acute/subacute form of PCM predominantly occurs in children and young adults with dominant Th2 and Th9 immunity and a limited or absent pulmonary inflammatory response to the pathogen. Consequently, there is prompt lymphatic–hematogenic dissemination of the fungus, and signs and symptoms may develop 3–12 weeks after exposure to *Paracoccidioides* spp. Acute PCM usually presents with generalized adenomegaly, mucocutaneous lesions, and hepatosplenomegaly [110,112].

In contrast, the chronic form of PCM is characterized by the reactivation of latent foci (mainly pulmonary), which usually occurs several decades after exposure to *Paracoccidioides* spp. This clinical form is much more common among men than among women of adult age (ratio 15–22:1), probably due to estrogen activity preventing the conversion of inhaled filamentous forms of the fungus into the pathogenic yeast form [113]. Lung lesions are present in 65–90% of chronic cases and may be combined with localized lymphadenopathy, mucocutaneous lesions, adrenal enlargement, and eventually central nervous system involvement. In addition to the immunological response of the host, the progression from infection to disease due to *Paracoccidioides* spp. may be modulated by genetic background. There is some evidence that the C4B*-Q0 antigen of the class III major histocompatibility complex and genotype IL12RB1 641 may be associated with the chronic disseminated form of this fungal disease. There are more robust data implicating lifestyle, especially smoking and alcohol consumption, in the progression of latent infection to fungal disease [59,86,114,115,116]. Indeed, a recent study in a mouse model of pulmonary PCM showed a negative effect of cigarette smoking on the local immune lung response to *Paracoccidioides* spp., making the animals more susceptible to pulmonary disease [117].

Lung lesions are secondary to the host response to pathogens that promote pulmonary fibrosis owing to persistent fungal antigen stimulation and excessive immune system activation. Once the tissue scarring process is triggered, it may progress even after the introduction of adequate treatment [59,118]. More recently, a study combining information that was generated by experimental mouse models and human data suggested that PCM-induced pulmonary hypertension may be influenced by the remodeling of the adventitial layer of pulmonary vessels and not only secondary to fibrosis [119].

Sequelae have mostly been documented in the lungs, but they may also affect the trachea, larynx, skin, adrenal glands, and central nervous system. Consequently, chronic respiratory insufficiency, dysphonia, Addison’s disease, and seizures are frequent findings that characterize the residual form of PCM [118,120,121,122].

### 3.3. Clinical Aspects

Pulmonary disease in PCM presents with an insidious course. Dyspnea and cough may persist for months to years before the diagnosis is made, and patients may also experience constitutional symptoms, such as anorexia, weight loss, and fever [56,111]. Notably, a clinical–radiological dissociation that is characterized by mild respiratory complaints among patients exhibiting extensive pulmonary involvement on radiological examination is not uncommon, and dyspnea is usually a late manifestation of pulmonary involvement [123,124]. Late diagnosis is usually related to several aspects, including insidious presentation of the disease, smoking-related chronic obstructive pulmonary disease, misdiagnosis of other chronic pulmonary conditions (mainly TB), limited access of rural workers to medical assistance, and lack of commercial diagnostic tests in routine laboratories of endemic regions [13].

Despite the low mortality rate that is usually attributed to PCM (1.17–8.2 per million inhabitants), patients frequently present with lung fibrosis, leading to severe restriction of respiratory function with a decline in their work capacity and quality of life [89,124,125].

Cohort studies that were conducted in the 1980s suggested that moderate-to-severe obstructive dysfunction may take place in more than 50% of patients with PCM after effective treatment [126,127]. Data provided by a single-center study conducted in Colombia revealed that the frequency of fibrosis after treatment was related to the extension of lung infiltrates at the time of diagnosis [118]. In this particular study, 45% of 47 patients evaluated remained with cough and/or dyspnea at the end of the planned antifungal therapy. Finally, emphysema and high initial serological titers for specific *Paracoccidioides* spp. antibodies (indicative of high fungal burden) were found to be variables independently associated with the severity of lung function impairment and a decrease in quality of life [124].

Considering the low specificity of signs and symptoms of pulmonary PCM, an important clue to consider in the clinical diagnosis of fungal disease is the concomitance of ulcerated or ulcer-vegetative lesions of the oral mucosa with characteristic hemorrhagic dots (moriform lesions), which may be present in 60% to 75% of patients with the chronic form of PCM. Skin lesions may also be found in 25% to 30% of patients, with a considerable polymorphism of manifestations, including papulonodular, ulcer-crusted, molluscoid, or acneiform lesions [85,86,128]. Notably, these lesions may facilitate laboratory diagnosis by direct mycological or histopathological examination of exudates or tissues [129]. When only pulmonary abnormalities are present, epidemiological inquiry and imaging findings represent the main red flags that trigger laboratory investigations of fungal infections.

### 3.4. Imaging Studies

**Figure 6:**
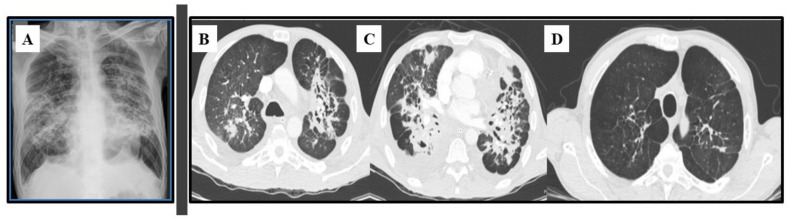
Lung images of a 53-year-old male patient with chronic paracoccidioidomycosis. (**A**) Plain chest radiograph with bilateral, parahilar, and symmetrical interstitial alveolar infiltrate (“butterfly wing” pattern); (**B**,**C**) Axial thoracic CT at diagnosis with multiple bronchiectasis associated with areas of irregular parenchymal opacities, and diffuse nodules (some of them excavated); (**D**) Axial thoracic CT at the end of antifungal therapy: areas of emphysema with traction bronchiectasis, retractable striae, and central architectural distortion, predominantly peribronchovascular, indicating fibrosis.

Plain chest radiographs classically indicate interstitial or mixed (alveolar interstitial) lesions, which are usually bilateral, perihilar, and symmetrical. PCM lesions are primarily located in the middle third of the lungs, resulting in the so-called “butterfly wing” pattern (see Figure 6A). Other findings include septum thickening, thick lines, alveolar opacities, fibrosis blocks, bronchial wall thickening, bronchiectasis, and cavities without fluid [130].

Advances in radiology techniques and the widespread use of computerized tomography (CT) have enabled the characterization of various patterns of pulmonary injuries (Figure 6B–D). Studies addressing CT findings in chronic PCM have described interlobular septal thickening as the most common alteration (90–96% of cases), followed by emphysema (60–70%) and ground-glass attenuation (58–67%). Bronchial wall thickening, nodules, cavitary nodules, cavities, pleural thickening, and parenchymatous bands have also been frequently reported [131,132]. These abnormalities are usually distributed in the posterior and peripheral regions of the lungs, with discrete predominance in the middle lung zone [133]. After treatment, signs of residual fibrosis persist in at least 30–40% of patients, such as architectural distortion (90%), reticulate and septal thickening (88%), centrilobular and paraseptal emphysema (84%), and parenchymal bands (74%) [118,124,134].

### 3.5. Laboratorial Diagnosis

**Figure 7:**
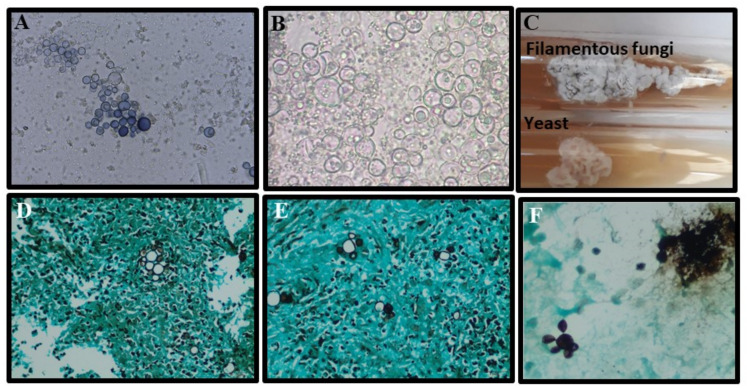
Morphological aspects of *Paracoccidioides* spp.: (**A**,**B**) Characteristic yeast forms showing multiple buddings (5 to 15 µm) seen upon direct examination of a sputum sample stained with a mix of KOH–Parker ink and only KOH, respectively; (**C**) *Paracoccidioides* spp. cultured on Sabouraud agar slants for 14 days, at 25 °C (top, filamentous phase) and 37 °C (underside, yeast phase); (**D**,**E**) Lung biopsy stained by Grocott methenamine silver stain showing yeasts with multiple buds (“pilot’s wheel” and “Mickey Mouse” elements); (**F**) Bronchoalveolar lavage stained with Grocott methenamine silver stain with yeast forms showing multiple budding (5 to 15 µm).

The gold standard method for PCM diagnosis is the isolation of *Paracoccidioides* spp. in culture, as well as microscopic visualization of typical yeast cells by direct mycological examination (DME) or histopathology [55,56] (Table 1). Direct mycological examination of exudates may be performed using 20–40% KOH solution, depending on the clinical sample, with or without Parker ink, by placing the sample between a slide and a coverslip [60,129]. Clinical samples for DME may be collected from skin or mucous membranes, sputum, bronchoalveolar lavage, and some difficult-to-access samples, such as biopsies from the lungs, the larynx, lymph nodes, central nervous system, and adrenals [135,136]. Typical fungal elements of *Paracoccidioides* spp. are represented by mother cells measuring 10–40 µm in diameter surrounded by multiple buds (blastoconidia) 2–6 µm in diameter, resembling the aspect of “pilot’s wheel” (multiple buds) or “Mickey Mouse” (two buds) [55,56,129]. Grocott’s methenamine silver and PAS stains provide better sensitivity when identifying fungal elements in tissue than HE [57,137]. The sensitivity and specificity of all conventional methods are summarized in Table 1. The morphological characteristics of *Paracoccidioides* spp. in culture, DME, and histopathology are shown in Figure 7.

Isolation of *Paracoccidioides* in culture has been performed mostly on Sabouraud and Mycosel agar, but other media may also be recommended, such as brain–heart infusion agar (BHI) and Sabouraud dextrose plus BHI broth (SABHI). *Paracoccidioides* is a fastidious fungus that grows slowly between 3 and 4 weeks, limiting its use for early diagnosis [55,56]. Like all thermodimorphic fungi, it grows at 25 °C as filamentous fungi and at 37 °C as yeast [56].

Concerning the recognition of *Paracoccidioides* species, several “in-house” PCR-based methods have been developed for the recognition of *Paracoccidioides* species, including nested PCR [138], quantitative real-time PCR [64], PCR-RFLP (restriction fragment length polymorphism) [139], and multilocus microsatellite typing (MLMT) [63]. However, the best method for *Paracoccidioides* spp. identification is still considered to be DNA sequencing followed by phylogenetic analysis. Considering that there is no commercially available molecular method for this identification, matrix-assisted laser desorption ionization time-of-flight mass spectrometry (MALDI-TOF) is a reliable and more suitable alternative for the accurate identification of *P. brasiliensis* complex and *P. Lutzii* in routine laboratories [55,129,140].

In clinical practice, histopathology and serological methods are the two most frequently used methods for diagnosing PCM in endemic areas [55,58]. Detection of specific *Paracoccidioides* spp. antibodies may be achieved by a large range of methods, including double immunodiffusion (DID), counterimmunoelectrophoresis (CIE), ELISA, immunoblotting, Western blotting, immunofluorescence, radioimmunoassay, dot immunoassay, and latex particle agglutination (LA) [58,141,142,143,144,145]. Notably, there is no single commercial test available for detecting specific *Paracoccidioides* spp. antibodies, and fungal antigens are prepared in-house and obtained from different strains of *Paracoccidioides* spp. Consequently, serological results may vary substantially from lot-to-lot preparation of antigens, as well as between different techniques, strains, and laboratories [60,144,146]. Assays for the detection of *P. brasiliensis* complex require standard exoantigen preparations containing high concentrations of GP43 glycoprotein, the immunodominant, and specific molecules in DID tests (the most commonly used) or 70 kDa glycoprotein [61,62,147]. To diagnose *P. lutzii* infections using the DID test, it is recommended to use cell-free antigens obtained from this particular species [62]. Besides providing a diagnosis, serological tests may also be used to monitor the host response to antifungal therapy [148]. The main characteristics of the serological tests are summarized in Table 1.

Cross-reactivity of PCM serological tests is common in patients with histoplasmosis and aspergillosis [144]. It is important to mention that immunocompromised patients with severe PCM may not produce antibodies and generate false-negative results [130,149].

Antigen detection assays and specific PCR methods have been developed by reference research centers but are not available in routine laboratories in most endemic regions. In this scenario, assays containing anti-gp43 and anti-gp47 monoclonal antibodies may be used to detect *Paracoccidioides* antigens in the serum, urine, sputum, cerebrospinal fluid, and bronchoalveolar lavage fluid [60]. Of note, patients with PCM may generate positive results for Glucan and Galactomannan detection in serum samples [149,150].

In recent decades, advances have been made in the development of new molecular techniques for the detection of *Paracoccidioides* DNA in clinical samples, including amplification of ribosomal DNA region (rDNA), gene-expressing GP43 glycoprotein, and quantitative Chain Reaction Polymerase (qPCR) platform duplex PCR assay [63,138,151,152]. The implementation of new methodologies based on photonic techniques and machine learning algorithms has been suggested, such as Fourier Transform Infrared (FTIR) and Raman spectroscopy [148,153,154].

### 3.6. Treatment

All *Paracoccidioides* spp. seem to respond equally to antifungals of different classes: Amph-B, azoles (ketoconazole, fluconazole, itraconazole, voriconazole, posaconazole, and isavuconazole), terbinafine, and sulfonamides.

The first therapeutic choice for mild and moderate forms of the disease is itraconazole (ITZ) 200 mg/day for 9–12 months, with response rates of 85–90% [155,156]. Unfortunately, the governments of endemic countries do not provide free ITZ to vulnerable populations with PCM, which makes it difficult to access this treatment [157]. The intravenous formulation of ITZ has not been commercialized in most PCM-endemic regions. Plasma-level monitoring of patients undergoing ITZ therapy has been recommended, but the vast majority of medical centers in Latin America are unable to do so [158]. To overcome this limitation, a novel formulation of ITZ with better bioavailability, labeled SUper BioAvailable (SUBA)-itraconazole, was developed [159]. However, a recent open-label comparative trial of SUBA versus conventional ITZ for the treatment of endemic mycoses showed almost identical plasma levels with similar specific adverse events in patients treated with both regimens. Notably, none of the PCM cases were included in this trial [160]. Despite all the limitations in monitoring plasma levels of ITZ in Latin America, considering that *Paracoccidioides* is highly susceptible to this drug, real-life experiences reporting outcomes of patients with PCM with ITZ are generally very good [161].

Trimethoprim–sulfamethoxazole (TMP/SMX) (160/800 mg bid or tid) has been widely used to treat PCM as an alternative to itraconazole. The TMP/SMX combination is found in oral and intravenous formulations and provides good absorption with predictable and stable plasma level concentrations and fewer drug–drug interactions than azoles. No randomized trials have evaluated the efficacy of TMP/SMX in comparison with that of ITZ in patients with PCM, but several retrospective studies have found that a longer treatment period is required with the TMP/SMX regimen (usually 2 years) when compared with ITZ treatment [155,161,162].

A 2- to 4-week course of Amph-B formulation is recommended for the initial treatment of severe cases of PCM [156,157]. Lipid formulations (3–5 mg/kg/day) are preferred over deoxycholate to avoid toxicity; however, they are costly, which limits access to them by most patients in endemic countries [157].

Second-generation azoles are therapeutic options with limited clinical experience in PCM treatment. Voriconazole showed similar efficacy to itraconazole in an open-label study with 53 patients in Brazil [163]. Isavuconazole was used to treat 10 Brazilian patients with disseminated disease, with partial success in 70% of cases and two patients progressing to death [164].

The most recent glucan synthase inhibitors (ibrexafungerp and rezafungin), as well as olorofilms, have not yet been evaluated for their in vitro or in vivo activity against PCM [165].

The time of treatment for PCM is usually defined individually, taking into consideration clinical and radiological improvement, as well as the decrease in antibody titers in those with a positive serology test at diagnosis [56,166,167].

### 3.7. New Perspectives in the Clinical Management

Several efforts have been made to develop a vaccine against PCM, but all of these are restricted to the preclinical stage of investigation [168]. Vaccination with peptide 10 (P10), derived from *P. brasiliensis* glycoprotein 43 (gp43), effectively reduced the fungal burden in the lungs, liver, and spleen in animal models. Several adjuvants have been tested, providing different ranges of adverse events and efficacy [169,170,171]. In addition to P10, immunization with the 27 kDa protein located at the surface and in the cytosol of *P. brasiliensis* has also been explored. Immunization with the 27 kDa protein may not only provide some protective effects but may also prevent pulmonary fibrosis in murine models of PCM [172].

Recently, several groups have explored strategies to mitigate pulmonary fibrosis secondary to antifungal therapy for PCM [173]. The antifibrotic activities of pentoxifylline (PTX), azithromycin (AZT), and thalidomide (Thal) in a murine model of pulmonary PCM treated with ITZ or cotrimoxazole (TMP/SMX) were investigated. The authors found that infected mice that were treated with PTX/ITC and AZT/CMX showed a reduction in the pulmonary concentrations of pro-inflammatory and pro-fibrotic factors [174].

Despite the relevance of neutrophils in the primary response to the fungal pathogen, evidence suggests that they may be detrimental during the chronic course of *Paracoccidioides* sp. infection, causing excessive tissue damage. Puerta-Portas et al. demonstrated that the combination of ITZ and a specific monoclonal antibody/mAb-anti-Ly6G used to deplete neutrophils could reduce the burden of infection and pulmonary fibrosis by downregulating inflammatory and pro-fibrotic genes [175]. These results are in line with those of a previous study demonstrating that depletion of neutrophils in the chronic phase of PCM in mice may substantially reduce fibrosis [176].

The use of monoclonal antibodies, alone or in combination with antifungal agents, has emerged as a potentially efficient treatment strategy to improve outcomes [177]. Boniche-Alfaro et al. [178] used a monoclonal antibody (mAbF1.4) against the cell wall glycoconjugate fraction of *Paracoccidioides* spp., combined with trimethoprim–sulfamethoxazole (TMP/SMX), to treat BALB/c mice with PCM. Using this combined therapy, the authors were able to obtain a significant reduction in pulmonary fungal burden, promoting a mixed Th1–Th17 type immune response that preserved the lung architecture [178].

Finally, in addition to developing strategies to modulate the host response to fungal pathogens without producing excessive fibrosis, it is mandatory to reinforce governmental initiatives to guarantee access to available diagnostic tools and medications for the most vulnerable rural patients with PCM.

## Data Availability

Not applicable.

## References

[1] Colombo A.L., Tobón A., Restrepo A., Queiroz-Telles F., Nucci M. (2011). Epidemiology of Endemic Systemic Fungal Infections in Latin America. Med. Mycol..

[2] Ashraf N., Kubat R.C., Poplin V., Adenis A.A., Denning D.W., Wright L., McCotter O., Schwartz I.S., Jackson B.R., Chiller T. (2020). Re-Drawing the Maps for Endemic Mycoses. Mycopathologia.

[3] Tirado-Sánchez A., González G.M., Bonifaz A. (2020). Endemic Mycoses: Epidemiology and Diagnostic Strategies. Expert Rev. Anti Infect. Ther..

[4] Johnson R.H., Sharma R., Kuran R., Fong I., Heidari A. (2021). Coccidioidomycosis: A Review. J. Investig. Med..

[5] Teixeira M.M., Theodoro R.C., de Carvalho M.J.A., Fernandes L., Paes H.C., Hahn R.C., Mendoza L., Bagagli E., San-Blas G., Felipe M.S.S. (2009). Phylogenetic Analysis Reveals a High Level of Speciation in the *Paracoccidioides* Genus. Mol. Phylogenet. Evol..

[6] Teixeira M.M., Cattana M.E., Matute D.R., Muñoz J.F., Arechavala A., Isbell K., Schipper R., Santiso G., Tracogna F., de los Ángeles Sosa M. (2020). Genomic Diversity of the Human Pathogen *Paracoccidioides* across the South American Continent. Fungal Genet. Biol..

[7] Hrycyk M.F., Garces H.G., Bosco S.D.M.G., de Oliveira S.L., Marques S.A., Bagagli E. (2018). Ecology of *Paracoccidioides brasiliensis*, *P. lutzii* and Related Species: Infection in Armadillos, Soil Occurrence and Mycological Aspects. Med. Mycol..

[8] Turissini D.A., Gomez O.M., Teixeira M.M., McEwen J.G., Matute D.R. (2017). Species Boundaries in the Human Pathogen *Paracoccidioides*. Fungal Genet. Biol..

[9] Vio de Queiroz-Telles F., Pietrobom P.M.P., Júnior M.R., Baptista R.D.M., Peçanha P.M. (2020). New Insights on Pulmonary Paracoccidioidomycosis. Semin. Respir. Crit. Care Med..

[10] Ekeng B.E., Davies A.A., Osaigbovo I.I., Warris A., Oladele R.O., Denning D.W. (2022). Pulmonary and Extrapulmonary Manifestations of Fungal Infections Misdiagnosed as Tuberculosis: The Need for Prompt Diagnosis and Management. J. Fungi.

[11] Matsuda J.D.S., Wanke B., Balieiro A.A.D.S., da Silva Santos C.S., Cavalcante R.C.D.S., de Medeiros Muniz M., Torres D.R., Pinheiro S.B., Frickmann H., Souza J.V.B. (2021). Prevalence of Pulmonary Mycoses in Smear-Negative Patients with Suspected Tuberculosis in the Brazilian Amazon. Rev. Iberoam Micol..

[12] Quagliato Júnior R., Grangeia T.D.A.G., Massucio R.A.D.C., de Capitani E.M., Rezende S.M., Balthazar A.B. (2007). Associação Entre Paracoccidioidomicose e Tuberculose: Realidade e Erro Diagnóstico. J. Brasil. Pneum..

[13] Griffiths J., Colombo A.L., Denning D.W. (2019). The Case for Paracoccidioidomycosis to Be Accepted as a Neglected Tropical (Fungal) Disease. PLoS Negl. Trop. Dis..

[14] Queiroz-Telles F., Fahal A.H., Falci D.R., Caceres D.H., Chiller T., Pasqualotto A.C. (2017). Neglected Endemic Mycoses. Lancet Infect. Dis..

[15] WHO (2022). WHO Fungal Priority Pathogens List to Guide Research, Development and Public Health Action.

[16] Bays D.J., Thompson G.R. (2021). Coccidioidomycosis. Infect. Dis. Clin. N. Am..

[17] Boro R., Iyer P.C., Walczak M.A. (2022). Current Landscape of Coccidioidomycosis. J. Fungi.

[18] Ampel N.M. (2009). Coccidioidomycosis: A Review of Recent Advances. Clin. Chest Med..

[19] Welsh O., Vera-Cabrera L., Rendon A., Gonzalez G., Bonifaz A. (2012). Coccidioidomycosis. Clin. Dermatol..

[20] Dickson E.C. (1937). “Valley Fever” of the San Joaquin Valley and Fungus Coccidioides. Cal. West Med..

[21] Crum N.F. (2022). Coccidioidomycosis: A Contemporary Review. Infect Dis. Ther..

[22] Chow N.A., Kangiser D., Gade L., McCotter O.Z., Hurst S., Salamone A., Wohrle R., Clifford W., Kim S., Salah Z. (2021). Factors Influencing Distribution of *Coccidioides immitis* in Soil, Washington State, 2016. mSphere.

[23] Williams S.L., Chiller T. (2022). Update on the Epidemiology, Diagnosis, and Treatment of Coccidioidomycosis. J. Fungi.

[24] Ramadan F.A., Ellingson K.D., Canales R.A., Bedrick E.J., Galgiani J.N., Donovan F.M. (2022). Cross-Sectional Study of Clinical Predictors of Coccidioidomycosis, Arizona, USA. Emerg. Infect. Dis..

[25] Carey A., Gorris M.E., Chiller T., Jackson B., Beadles W., Webb B.J. (2021). Epidemiology, Clinical Features, and Outcomes of Coccidioidomycosis, Utah, 2006–2015. Emerg. Infect. Dis..

[26] McCotter O.Z., Benedict K., Engelthaler D.M., Komatsu K., Lucas K.D., Mohle-Boetani J.C., Oltean H., Vugia D., Chiller T.M., Cooksey G.L.S. (2019). Update on the Epidemiology of Coccidioidomycosis in the United States. Med. Mycol..

[27] Ampel N.M. (2010). What’s Behind the Increasing Rates of Coccidioidomycosis in Arizona and California?. Curr. Infect. Dis. Rep..

[28] Baptista Rosas R.C., Riquelme M. (2007). Epidemiología de La Coccidioidomicosis En México. Rev Iberoam Micol.

[29] Laniado-Laborín R., Arathoon E.G., Canteros C., Muñiz-Salazar R., Rendon A. (2019). Coccidioidomycosis in Latin America. Med. Mycol..

[30] Canteros C.E., Toranzo A., Ibarra-Camou B., David V., Carrizo S.G., Santillán-Iturres A., Serrano J., Fernández N., Capece P., Gorostiaga J. (2010). Coccidioidomycosis in Argentina, 1892-2009. Rev. Argent Microbiol..

[31] Cordeiro R., Moura S., Castelo-Branco D., Rocha M.F., Lima-Neto R., Sidrim J.J. (2021). Coccidioidomycosis in Brazil: Historical Challenges of a Neglected Disease. J. Fungi.

[32] Diaz J.H. (2018). Travel-Related Risk Factors for Coccidioidomycosis. J. Travel Med..

[33] Carpenter J.B., Feldman J.S., Leyva W.H., DiCaudo D.J. (2010). Clinical and Pathologic Characteristics of Disseminated Cutaneous Coccidioidomycosis. J. Am. Acad. Dermatol..

[34] Grizzle A.J., Wilson L., Nix D.E., Galgiani J.N. (2021). Clinical and Economic Burden of Valley Fever in Arizona: An Incidence-Based Cost-of-Illness Analysis. Open Forum Infect. Dis..

[35] Del Rocío Reyes-Montes M., Pérez-Huitrón M.A., Ocaña-Monroy J.L., Frías-De-León M.G., Martínez-Herrera E., Arenas R., Duarte-Escalante E. (2016). The Habitat of *Coccidioides* spp. and the Role of Animals as Reservoirs and Disseminators in Nature. BMC Infect. Dis..

[36] Kirkland T.N., Fierer J. (2018). *Coccidioides immitis* and *posadasii*; A Review of Their Biology, Genomics, Pathogenesis, and Host Immunity. Virulence.

[37] Huppert M., Sun S.H., Harrison J.L. (1982). Morphogenesis throughout Saprobic and Parasitic Cycles of *Coccidioides immitis*. Mycopathologia.

[38] Blair J.E., Chang Y.-H.H., Cheng M.-R., Vaszar L.T., Vikram H.R., Orenstein R., Kusne S., Ho S., Seville M.T., Parish J.M. (2014). Characteristics of Patients with Mild to Moderate Primary Pulmonary Coccidioidomycosis. Emerg. Infect. Dis..

[39] Spinello I., Munoz A., Johnson R. (2008). Pulmonary Coccidioidomycosis. Semin. Respir. Crit. Care Med..

[40] Stockamp N.W., Thompson G.R. (2016). Coccidioidomycosis. Infect. Dis. Clin. N. Am..

[41] Nguyen C., Barker B.M., Hoover S., Nix D.E., Ampel N.M., Frelinger J.A., Orbach M.J., Galgiani J.N. (2013). Recent Advances in Our Understanding of the Environmental, Epidemiological, Immunological, and Clinical Dimensions of Coccidioidomycosis. Clin. Microbiol. Rev..

[42] Cadena J., Hartzler A., Hsue G., Longfield R.N. (2009). Coccidioidomycosis and Tuberculosis Coinfection at a Tuberculosis Hospital. Medicine.

[43] Galgiani J.N., Ampel N.M., Blair J.E., Catanzaro A., Geertsma F., Hoover S.E., Johnson R.H., Kusne S., Lisse J., MacDonald J.D. (2016). 2016 Infectious Diseases Society of America (IDSA) Clinical Practice Guideline for the Treatment of Coccidioidomycosis. Clin. Infect. Dis..

[44] Garcia Garcia S.C., Salas Alanis J.C., Flores M.G., Gonzalez Gonzalez S.E., Vera Cabrera L., Ocampo Candiani J. (2015). Coccidioidomycosis and the Skin: A Comprehensive Review. An. Bras. Dermatol..

[45] Jackson N.R., Blair J.E., Ampel N.M. (2019). Central Nervous System Infections Due to Coccidioidomycosis. J. Fungi.

[46] Heaney A.K., Head J.R., Broen K., Click K., Taylor J., Balmes J.R., Zelner J., Remais J.V. (2021). Coccidioidomycosis and COVID-19 Co-Infection, United States, 2020. Emerg. Infect. Dis..

[47] Huff D., Ampel N.M., Blair J.E. (2022). Coccidioidomycosis and COVID-19 Infection. An Analysis from a Single Medical Center Within the Coccidioidal Endemic Area. Mycopathologia.

[48] Aly F.Z., Millius R., Sobonya R., Aboul-Nasr K., Klein R. (2016). Cytologic Diagnosis of Coccidioidomycosis: Spectrum of Findings in Southern Arizona Patients over a 10 Year Period. Diagn Cytopathol.

[49] Grosse A., Grosse C. (2019). Coccidioidomycosis with Emperipolesis in Fine Needle Aspiration. Cytopathology.

[50] Gastélum-Cano J.M., Dautt-Castro M., García-Galaz A., Felix-Murray K., Rascón-Careaga A., Cano-Rangel M.A., Islas-Osuna M.A. (2021). The Clinical Laboratory Evolution in Coccidioidomycosis Detection: Future Perspectives. J. Med. Mycol..

[51] Gabe L.M., Malo J., Knox K.S. (2017). Diagnosis and Management of Coccidioidomycosis. Clin. Chest Med..

[52] Malo J., Luraschi-Monjagatta C., Wolk D.M., Thompson R., Hage C.A., Knox K.S. (2014). Update on the Diagnosis of Pulmonary Coccidioidomycosis. Ann. Am. Thorac. Soc..

[53] Binnicker M.J., Buckwalter S.P., Eisberner J.J., Stewart R.A., McCullough A.E., Wohlfiel S.L., Wengenack N.L. (2007). Detection of *Coccidioides* Species in Clinical Specimens by Real-Time PCR. J. Clin. Microbiol..

[54] Umeyama T., Sano A., Kamei K., Niimi M., Nishimura K., Uehara Y. (2006). Novel Approach to Designing Primers for Identification and Distinction of the Human Pathogenic Fungi *Coccidioides immitis* and *Coccidioides posadasii* by PCR Amplification. J. Clin. Microbiol..

[55] Hahn R.C., Hagen F., Mendes R.P., Burger E., Nery A.F., Siqueira N.P., Guevara A., Rodrigues A.M., de Camargo Z.P. (2022). Paracoccidioidomycosis: Current Status and Future Trends. Clin. Microbiol. Rev..

[56] Shikanai-Yasuda M.A., Mendes R.P., Colombo A.L., de Queiroz-Telles F., Kono A.S.G., Paniago A.M.M., Nathan A., do Valle A.C.F., Bagagli E., Benard G. (2017). Brazilian Guidelines for the Clinical Management of Paracoccidioidomycosis. Rev. Soc. Bras. Med. Trop..

[57] Dutra L.M., Silva T.H.M., Falqueto A., Peçanha P.M., Souza L.R.M., Gonçalves S.S., Velloso T.R.G. (2018). Oral Paracoccidioidomycosis in a Single-Center Retrospective Analysis from a Brazilian Southeastern Population. J. Infect. Public Health.

[58] de Camargo Z.P. (2008). Serology of Paracoccidioidomycosis. Mycopathologia.

[59] Benard G. (2008). An Overview of the Immunopathology of Human Paracoccidioidomycosis. Mycopathologia.

[60] Da Silva S.H.M., Colombo A.L., Blotta M.H.S.L., Lopes J.D., Queiroz-Telles F., de Camargo Z.P. (2003). Detection of Circulating Gp43 Antigen in Serum, Cerebrospinal Fluid, and Bronchoalveolar Lavage Fluid of Patients with Paracoccidioidomycosis. J. Clin. Microbiol..

[61] De Mattos Grosso D., de Almeida S.R., Mariano M., Lopes J.D. (2003). Characterization of Gp70 and Anti-Gp70 Monoclonal Antibodies in *Paracoccidioides brasiliensis* Pathogenesis. Infect. Immun..

[62] Gegembauer G., Araujo L.M., Pereira E.F., Rodrigues A.M., Paniago A.M.M., Hahn R.C., Camargo Z.P.D. (2014). Serology of Paracoccidioidomycosis Due to *Paracoccidioides lutzii*. PLoS Negl. Trop. Dis..

[63] Pinheiro B.G., Hahn R.C., Camargo Z.P.D., Rodrigues A.M. (2020). Molecular Tools for Detection and Identification of *Paracoccidioides* Species: Current Status and Future Perspectives. J. Fungi.

[64] Rocha-Silva F., Gomes L.I., Gracielle-Melo C., Goes A.M., Caligiorne R.B. (2017). Real Time Polymerase Chain Reaction (Rt-PCR): A New Patent to Diagnostic Purposes for Paracoccidioidomycosis. Recent Pat. Endocr. Metab. Immune Drug Discov..

[65] Ampel N.M. (2010). The Diagnosis of Coccidioidomycosis. F1000 Med. Rep..

[66] Vucicevic D., Blair J.E., Binnicker M.J., McCullough A.E., Kusne S., Vikram H.R., Parish J.M., Wengenack N.L. (2010). The Utility of *Coccidioides* Polymerase Chain Reaction Testing in the Clinical Setting. Mycopathologia.

[67] Kaufman L., Sekhon A.S., Moledina N., Jalbert M., Pappagianis D. (1995). Comparative Evaluation of Commercial Premier EIA and Microimmunodiffusion and Complement Fixation Tests for *Coccidioides immitis* Antibodies. J. Clin. Microbiol..

[68] Durkin M., Connolly P., Kuberski T., Myers R., Kubak B.M., Bruckner D., Pegues D., Wheat L.J. (2008). Diagnosis of Coccidioidomycosis with Use of the *Coccidioides* Antigen Enzyme Immunoassay. Clin. Infect. Dis..

[69] Thompson G.R., Boulware D.R., Bahr N.C., Clancy C.J., Harrison T.S., Kauffman C.A., Le T., Miceli M.H., Mylonakis E., Nguyen M.H. (2022). Noninvasive Testing and Surrogate Markers in Invasive Fungal Diseases. Open Forum Infect. Dis..

[70] Saubolle M.A. (2007). Laboratory Aspects in the Diagnosis of Coccidioidomycosis. Ann. N. Y. Acad. Sci..

[71] Kim M.M., Vikram H.R., Kusne S., Seville M.T., Blair J.E. (2011). Treatment of Refractory Coccidioidomycosis With Voriconazole or Posaconazole. Clin. Infect. Dis..

[72] Shubitz L.F., Roy M.E., Nix D.E., Galgiani J.N. (2013). Efficacy of Nikkomycin Z for Respiratory Coccidioidomycosis in Naturally Infected Dogs. Med. Mycol..

[73] Sass G., Larwood D.J., Martinez M., Shrestha P., Stevens D.A. (2021). Efficacy of Nikkomycin Z in Murine CNS Coccidioidomycosis: Modelling Sustained-Release Dosing. J. Antimicrob. Chemother..

[74] Larwood D.J. (2020). Nikkomycin Z—Ready to Meet the Promise?. J. Fungi.

[75] Sass G., Larwood D.J., Martinez M., Chatterjee P., Xavier M.O., Stevens D.A. (2021). Nikkomycin Z against Disseminated Coccidioidomycosis in a Murine Model of Sustained-Release Dosing. Antimicrob. Agents Chemother..

[76] Wiederhold N.P. (2020). Review of the Novel Investigational Antifungal Olorofim. J. Fungi.

[77] Wiederhold N.P., Najvar L.K., Jaramillo R., Olivo M., Birch M., Law D., Rex J.H., Catano G., Patterson T.F. (2018). The Orotomide Olorofim Is Efficacious in an Experimental Model of Central Nervous System Coccidioidomycosis. Antimicrob. Agents Chemother..

[78] Pappagianis D., Levine H.B. (1975). The Present Status of Vaccination Against Coccidioidomycosis in Man. Am. J. Epidemiol..

[79] Cole G.T., Xue J.M., Okeke C.N., Tarcha E.J., Basrur V., Schaller R.A., Herr R.A., Yu J.J., Hung C.Y. (2004). Prospects of Vaccines for Medically Important Fungi. A Vaccine against Coccidioidomycosis Is Justified and Attainable. Med. Mycol..

[80] Galgiani J.N., Shubitz L.F., Orbach M.J., Mandel M.A., Powell D.A., Klein B.S., Robb E.J., Ohkura M., Seka D.J., Tomasiak T.M. (2022). Vaccines to Prevent Coccidioidomycosis: A Gene-Deletion Mutant of *Coccidioides posadasii* as a Viable Candidate for Human Trials. J. Fungi.

[81] Martinez R. (2017). New Trends in Paracoccidioidomycosis Epidemiology. J. Fungi.

[82] Mavengere H., Mattox K., Teixeira M.M., Sepúlveda V.E., Gomez O.M., Hernandez O., McEwen J., Matute D.R. (2020). Paracoccidioides Genomes Reflect High Levels of Species Divergence and Little Interspecific Gene Flow. mBio.

[83] Roberto T.N., de Carvalho J.A., Beale M.A., Hagen F., Fisher M.C., Hahn R.C., de Camargo Z.P., Rodrigues A.M. (2021). Trends in the Molecular Epidemiology and Population Genetics of Emerging *Sporothrix* Species. Stud. Mycol..

[84] Bellissimo-Rodrigues F., Machado A.A., Martinez R. (2011). Paracoccidioidomycosis Epidemiological Features of a 1,000-Cases Series from a Hyperendemic Area on the Southeast of Brazil. Am. J. Trop. Med. Hyg..

[85] Peçanha P.M., Batista Ferreira M.E., Massaroni Peçanha M.A., Schmidt E.B., Lamas de Araújo M., Zanotti R.L., Potratz F.F., Delboni Nunes N.E., Gonçalves Ferreira C.U., Delmaestro D. (2017). Paracoccidioidomycosis: Epidemiological and Clinical Aspects in 546 Cases Studied in the State of Espírito Santo, Brazil. Am. J. Trop. Med. Hyg..

[86] Bellissimo-Rodrigues F., Bollela V.R., Fonseca B.A.L., Martinez R. (2013). Endemic Paracoccidioidomycosis: Relationship between Clinical Presentation and Patients’ Demographic Features. Med. Mycol..

[87] Coutinho Z.F., da Silva D., Lazéra M., Petri V., de Oliveira R.M., Sabroza P.C., Wanke B. (2002). Paracoccidioidomycosis Mortality in Brazil (1980-1995). Cad Saude Publica.

[88] Coutinho Z.F., Wanke B., Travassos C., Oliveira R.M., Xavier D.R., Coimbra C.E.A. (2015). Hospital Morbidity Due to Paracoccidioidomycosis in Brazil (1998–2006). Trop. Med. Int. Health.

[89] De Suguiura I.M.S., Ono M.A. (2022). Compulsory Notification of Paracoccidioidomycosis: A 14-Year Retrospective Study of the Disease in the State of Paraná, Brazil. Mycoses.

[90] Dantas K.C., Mauad T., de André C.D.S., Bierrenbach A.L., Saldiva P.H.N. (2021). A Single-Centre, Retrospective Study of the Incidence of Invasive Fungal Infections during 85 Years of Autopsy Service in Brazil. Sci. Rep..

[91] Fabris L.R., Andrade Ú.V., Santos A.F.D., Marques A.P.D.C., Oliveira S.M.D.V.L.D., Mendes R.P., Paniago A.M.M. (2014). Decreasing Prevalence of the Acute/Subacute Clinical Form of Paracoccidioidomycosis in Mato Grosso do Sul State, Brazil. Rev. Inst. Med. Trop. Sao Paulo.

[92] Vieira G.D.D., Alves T.D.C., Lima S.M.D.D., Camargo L.M.A., Sousa C.M.D. (2014). Paracoccidioidomycosis in a Western Brazilian Amazon State: Clinical-Epidemiologic Profile and Spatial Distribution of the Disease. Rev. Soc. Bras. Med. Trop..

[93] Krakhecke-Teixeira A.G., Yamauchi D.H., Rossi A., de Sousa H.R., Garces H.G., Júnior J.L., Júnior A.O.S., Felipe M.S.S., Bagagli E., de Andrade H.F. (2022). Clinical and Eco-Epidemiological Aspects of a Novel Hyperendemic Area of Paracoccidioidomycosis in the Tocantins-Araguaia Basin (Northern Brazil), Caused by *Paracoccidioides* sp.. J. Fungi.

[94] Mangiaterra M.L., Giusiano G.E., Alonso J.M., Gorodner J.O. (1999). *Paracoccidioides brasiliensis* Infection in a Subtropical Region with Important Environmental Changes. Bull. Soc. Pathol. Exot..

[95] Do Valle A.C.F., de Macedo P.M., Almeida-Paes R., Romão A.R., Lazéra M.D.S., Wanke B. (2017). Paracoccidioidomycosis after Highway Construction, Rio de Janeiro, Brazil. Emerg. Infect. Dis..

[96] Barrozo L.V., Benard G., Silva M.E.S., Bagagli E., Marques S.A., Mendes R.P. (2010). First Description of a Cluster of Acute/Subacute Paracoccidioidomycosis Cases and Its Association with a Climatic Anomaly. PLoS Negl. Trop. Dis..

[97] Giusiano G., Aguirre C., Vratnica C., Rojas F., Corallo T., Cattana M.E., Fernández M., Mussin J., de los Angeles Sosa M. (2019). Emergence of Acute/Subacute Infant-Juvenile Paracoccidioidomycosis in Northeast Argentina: Effect of Climatic and Anthropogenic Changes?. Med. Mycol..

[98] Wagner G., Moertl D., Glechner A., Mayr V., Klerings I., Zachariah C., den Nest M., Gartlehner G., Willinger B. (2021). Paracoccidioidomycosis Diagnosed in Europe—A Systematic Literature Review. J. Fungi.

[99] Rahman R., Davies L., Mohareb A.M., Peçanha-Pietrobom P.M., Patel N.J., Solomon I.H., Meredith D.M., Tsai H.K., Guenette J.P., Bhattacharyya S. (2020). Delayed Relapse of Paracoccidioidomycosis in the Central Nervous System: A Case Report. Open Forum Infect Dis..

[100] Onda H., Komine M., Murata S., Ohtsuki M. (2011). Letter: Imported Paracoccidioidomycosis in Japan. Dermatol. Online J..

[101] Linares G., Baker R.D., Linares L. (1971). Paracoccidioidomycosis in the United States (South American Blastomycosis). Arch. Otolaryngol. -Head Neck Surg..

[102] Falcão E.M., de Macedo P.M., Freitas D.F.S., Freitas A.D.A., Grinsztejn B., Veloso V.G., Almeida-Paes R., do Valle A.C.F. (2022). Paracoccidioidomycosis in People Living with HIV/AIDS: A Historical Retrospective Cohort Study in a National Reference Center for Infectious Diseases, Rio de Janeiro, Brazil. PLoS Negl. Trop. Dis..

[103] Morejón K.M.L., Machado A.A., Martinez R. (2009). Paracoccidioidomycosis in Patients Infected with and Not Infected with Human Immunodeficiency Virus: A Case-Control Study. Am. J. Trop. Med. Hyg..

[104] Messina F., Romero M., Benchetrit A., Marin E., Arechavala A., Depardo R., Negroni R., Santiso G. (2020). Clinical and Microbiological Characteristics of Paracoccidioidomycosis in Patients with AIDS in Buenos Aires, Argentina. Med. Mycol..

[105] De Almeida J., Peçanha-Pietrobom P., Colombo A. (2018). Paracoccidioidomycosis in Immunocompromised Patients: A Literature Review. J. Fungi.

[106] Valentim F.D.O., Tsutsui G.M., Abbade L.P.F., Marques S.A. (2021). Disseminated Paracoccidioidomycosis in a Liver Transplant Patient. An. Bras. Dermatol..

[107] Peçanha-Pietrobom P.M., Falqueto A., Rodrigues Gandarella A.D., Moyzés J.V., Rangel K.A., Miranda L.B., Hemerly M.C., Careta R.S., Peçanha P.M. (2019). Case Report: Paracoccidioidomycosis in Solid Organ Transplantation: Disseminated Disease in a Liver Recipient and Literature Review. Am. J. Trop. Med. Hyg..

[108] Felipe C.R.A., Silva A.D., Penido M.G.M.G. (2021). Disseminated Paracoccidioidomycosis in a Kidney Transplant Recipient. Cureus.

[109] Pereira R.M., Bucaretchi F., Barison E.D.M., Hessel G., Tresoldi A.T. (2004). Paracoccidioidomycosis in Children: Clinical Presentation, Follow-up and Outcome. Rev. Inst. Med. Trop. Sao Paulo.

[110] Benard G., Kavakama J., Mendes-Giannini M.J.S., Kono A., Duarte A.J.S., Shikanai-Yasuda M.A. (2005). Contribution to the Natural History of Paracoccidioidomycosis: Identification of the Primary Pulmonary Infection in the Severe Acute Form of the Disease–A Case Report. Clin. Infect. Dis..

[111] Mendes R.P., Cavalcante R.S., Marques S.A., Marques M.E.A., Venturini J., Sylvestre T.F., Paniago A.M.M., Pereira A.C., da Silva J.D.F., Fabro A.T. (2017). Paracoccidioidomycosis: Current Perspectives from Brazil. Open Microbiol. J..

[112] De Macedo P.M., Almeida-Paes R., Freitas D.F.S., Varon A.G., Paixão A.G., Romão A.R., Coutinho Z.F., Pizzini C.V., Zancopé-Oliveira R.M., do Valle A.C.F. (2017). Acute Juvenile Paracoccidioidomycosis: A 9-Year Cohort Study in the Endemic Area of Rio de Janeiro, Brazil. PLoS Negl. Trop. Dis..

[113] Restrepo A., Salazar M.E., Cano L.E., Stover E.P., Feldman D., Stevens D.A. (1984). Estrogens Inhibit Mycelium-to-Yeast Transformation in the Fungus *Paracoccidioides brasiliensis*: Implications for Resistance of Females to Paracoccidioidomycosis. Infect. Immun..

[114] Carvalho F.M.C., Busser F.D., Freitas V.L.T., Furucho C.R., Sadahiro A., Kono A.S.G., Criado P.R., Moretti M.L., Sato P.K., Shikanai-Yasuda M.A. (2016). Polymorphisms on IFNG, IL12B and IL12RB1 Genes and Paracoccidioidomycosis in the Brazilian Population. Infect. Genet. Evol..

[115] Dos Santos W.A., da Silva B.M., Passos E.D., Zandonade E., Falqueto A. (2003). Associação Entre Tabagismo e Paracoccidioidomicose: Um Estudo de Caso-Controle No Estado Do Espírito Santo, Brasil. Cad. Saude Publica.

[116] De Messias I.J.T., Reis A., Brenden M., Queiroz-Telles F., Mauff G. (1991). Association of Major Histocompatibility Complex Class III Complement Components C2, BF, and C4 with Brazilian Paracoccidioidomycosis. Complement. Inflamm..

[117] Buccheri R., Duarte-Neto A.N., Silva F.L.B., Haddad G.C., da Silva L.B.R., Netto R.A., Ledesma F.L., Taborda C.P., Benard G. (2022). Chronic Exposure to Cigarette Smoke Transiently Worsens the Disease Course in a Mouse Model of Pulmonary Paracoccidioidomycosis. Rev. Inst. Med. Trop. Sao Paulo.

[118] Tobon A.M., Agudelo C.A., Osorio M.L., Alvarez D.L., Arango M., Cano L.E., Restrepo A. (2003). Residual Pulmonary Abnormalities in Adult Patients with Chronic Paracoccidioidomycosis: Prolonged Follow-Up after Itraconazole Therapy. Clin. Infect. Dis..

[119] Batah S.S., Alda M.A., Machado-Rugulo J.R., Felix R.G., Nascimento E., Martinez R., Pádua A.I., Bagagli E., Hrycyk M.F., Salgado H.C. (2020). Pulmonary Paracoccidioidomycosis-induced Pulmonary Hypertension. Clin. Transl. Med..

[120] Colombo A.L., Faiçal S., Kater C.E. (1994). Systematic Evaluation of the Adrenocortical Function in Patients with Paracoccidioidomycosis. Mycopathologia.

[121] Francesconi F., da Silva M.T.T., Costa R.L.B., Francesconi V.A., Carregal E., Talhari S., do Valle A.C.F. (2011). Long-Term Outcome of Neuroparacoccidioidomycosis Treatment. Rev. Soc. Bras. Med. Trop..

[122] Alvarez M., Pina D.R., de Oliveira M., Ribeiro S.M., Mendes R.P., Duarte S.B., Miranda J.R.A. (2014). Objective CT-Based Quantification of Lung Sequelae in Treated Patients With Paracoccidioidomycosis. Medicine.

[123] Tobon A.M., Agudelo C.A., Restrepo C.A., Villa C.A., Quiceno W., Estrada S., Restrepo A. (2010). Adrenal Function Status in Patients with Paracoccidioidomycosis After Prolonged Post-Therapy Follow Up. Am. Trop. Med. Hyg..

[124] Costa A.N., Benard G., Albuquerque A.L.P., Fujita C.L., Magri A.S.K., Salge J.M., Shikanai-Yasuda M.A., Carvalho C.R.R. (2013). The Lung in Paracoccidioidomycosis: New Insights into Old Problems. Clinics.

[125] Prado M., da Silva M.B., Laurenti R., Travassos L.R., Taborda C.P. (2009). Mortality Due to Systemic Mycoses as a Primary Cause of Death or in Association with AIDS in Brazil: A Review from 1996 to 2006. Mem. Inst. Oswaldo Cruz..

[126] Campos E.P., Padovani C.R., Cataneo A.M. (1991). Paracoccidioidomycosis: Radiologic and Pulmonary Study in 58 Cases. Rev. Inst. Med. Trop. Sao Paulo.

[127] Campos E.P., Cataneo A.J.M. (1986). Função Pulmonar Na Evolução de 35 Doentes Com Paracoccidioidomicose. Rev. Inst. Med. Trop. Sao Paulo.

[128] Marques S.A., Cortez D.B., Lastória J.C., Camargo R.M.P.D., Marques M.E.A. (2007). Paracoccidioidomicose: Frequência, Morfologia e Patogênese de Lesões Tegumentares. An. Bras. Dermatol..

[129] Peçanha P.M., Peçanha-Pietrobom P.M., Grão-Velloso T.R., Rosa Júnior M., Falqueto A., Gonçalves S.S. (2022). Paracoccidioidomycosis: What We Know and What Is New in Epidemiology, Diagnosis, and Treatment. J. Fungi.

[130] Rosa Júnior M., Baldon I.V., Amorim A.F.C., Fonseca A.P.A., Volpato R., Lourenço R.B., Baptista R.M., de Mello R.A.F., Peçanha P., Falqueto A. (2018). Imaging Paracoccidioidomycosis: A Pictorial Review from Head to Toe. Eur. J. Radiol..

[131] Marchiori E., Valiante P.M., Mano C.M., Zanetti G., Escuissato D.L., Souza A.S., Capone D. (2011). Paracoccidioidomycosis: High-Resolution Computed Tomography–Pathologic Correlation. Eur. J. Radiol..

[132] Muniz M., Marchiori E., Magnago M., Moreira L. (2002). Paracoccidioidomicose Pulmonar: Aspectos Na Tomografia Computadorizada de Alta Resolução. Radiol. Bras..

[133] Souza A.S., Gasparetto E.L., Davaus T., Escuissato D.L., Marchiori E. (2006). High-Resolution CT Findings of 77 Patients with Untreated Pulmonary Paracoccidioidomycosis. Am. J. Roentg..

[134] De Pina D.R., Alvarez M., Giacomini G., Pavan A.L.M., Guedes C.I.A., de Souza Cavalcante R., Mendes R.P., Paniago A.M.M. (2017). Paracoccidioidomycosis: Level of Pulmonary Sequelae in High Resolution Computed Tomography Images from Patients of Two Endemic Regions of Brazil. Quant. Imaging Med. Surg..

[135] Restrepo A., Benard G., de Castro C., Agudelo C., Tobón A. (2008). Pulmonary Paracoccidioidomycosis. Semin. Respir. Crit. Care Med..

[136] Queiroz-Telles F., Escuissato D. (2011). Pulmonary Paracoccidioidomycosis. Semin. Respir. Crit. Care Med..

[137] Talhari C., de Souza J.V.B., Parreira V.J., Reinel D., Talhari S. (2008). Oral Exfoliative Cytology as a Rapid Diagnostic Tool for Paracoccidioidomycosis. Mycoses.

[138] Gaviria M., Rivera V., Muñoz-Cadavid C., Cano L.E., Naranjo T.W. (2015). Validation and Clinical Application of a Nested PCR for Paracoccidioidomycosis Diagnosis in Clinical Samples from Colombian Patients. Braz. J. Infec. Dis..

[139] Niño-Vega G.A., Calcagno A.M., San-Blas G., San-Blas F., Gooday G.W., Gow N.A.R. (2000). RFLP Analysis Reveals Marked Geographical Isolation between Strains of *Paracoccidioides brasiliensis*. Med. Mycol..

[140] Nobrega de Almeida J., del Negro G.M.B., Grenfell R.C., Vidal M.S.M., Thomaz D.Y., de Figueiredo D.S.Y., Bagagli E., Juliano L., Benard G. (2015). Matrix-Assisted Laser Desorption Ionization–Time of Flight Mass Spectrometry for Differentiation of the Dimorphic Fungal Species *Paracoccidioides brasiliensis* and *Paracoccidioides lutzii*. J. Clin. Microbiol.

[141] Camargo Z.P., Berzaghi R., Amaral C.C., Silva S.H.M. (2003). Simplified Method for Producing *Paracoccidioides brasiliensis* Exoantigens for Use in Immunodiffusion Tests. Med. Mycol..

[142] Restrepo A., Moncada L.H. (1978). A Slide Latex Test for the Diagnosis of Paracoccidiodomycosis. Bol. Oficina Sanit Panam.

[143] Silveira-Gomes F., Sarmento D.N., Pinto T.M., Pimentel R.F., Nepomuceno L.B., Santo E.P.T.E., Mesquita-da-Costa M., Camargo Z.P., Marques-da-Silva S.H. (2011). Development and Evaluation of a Latex Agglutination Test for the Serodiagnosis of Paracoccidioidomycosis. Clin. Vaccine Immunol..

[144] Teles F.R.R., Martins M.L. (2011). Laboratorial Diagnosis of Paracoccidioidomycosis and New Insights for the Future of Fungal Diagnosis. Talanta.

[145] Mendes-Giannini M.J., Bueno J.P., Shikanai-Yasuda M.A., Ferreira A.W., Masuda A. (1989). Detection of the 43,000-Molecular-Weight Glycoprotein in Sera of Patients with Paracoccidioidomycosis. J. Clin. Microbiol..

[146] Vidal M.S.M., Negro G.M.B., Vicentini A.P., Svidzinski T.I.E., Mendes-Giannini M.J., Almeida A.M.F., Martinez R., de Camargo Z.P., Taborda C.P., Benard G. (2014). Serological Diagnosis of Paracoccidioidomycosis: High Rate of Inter-Laboratorial Variability among Medical Mycology Reference Centers. PLoS Negl. Trop. Dis..

[147] Maricato J.T., Batista W.L., Kioshima É.S., Feitosa L.S., e Brito R.R.N., Goldman G.H., Mariano M., Puccia R., Lopes J.D. (2010). The *Paracoccidioides brasiliensis* Gp70 Antigen Is Encoded by a Putative Member of the Flavoproteins Monooxygenase Family. Fungal Genet. Biol..

[148] De Brito E.C.A., Franca T., Canassa T., Weber S.S., Paniago A.M.M., Cena C. (2022). Paracoccidioidomycosis Screening Diagnosis by FTIR Spectroscopy and Multivariate Analysis. Photodiagnosis. Photodyn. Ther..

[149] Xavier M.O., Pasqualotto A.C., Cardoso I.C.E., Severo L.C. (2009). Cross-Reactivity of *Paracoccidioides brasiliensis*, *Histoplasma capsulatum*, and *Cryptococcus* Species in the Commercial Platelia *Aspergillus* Enzyme Immunoassay. Clin. Vaccine Immunol..

[150] Melo A.S.D.A., Santos D.W.D.C.L., Lima S.L., Rodrigues A.M., Camargo Z.P., Finkelman M., Colombo A.L. (2020). Evaluation of (1 → 3)-β-D-glucan Assay for Diagnosing Paracoccidioidomycosis. Mycoses.

[151] Koishi A.C., Vituri D.F., Filho P.S.R.D., Sasaki A.A., Felipe M.S.S., Venancio E.J. (2010). A Semi-Nested PCR Assay for Molecular Detection of *Paracoccidioides brasiliensis* in Tissue Samples. Rev. Soc. Bras. Med. Trop..

[152] Imai T., Sano A., Mikami Y., Watanabe K., Aoki F.H., Branchini M.L.M., Negroni R., Nishimura K., Miyaji M. (2000). A New PCR Primer for the Identification of *Paracoccidioides Brasiliensis* Based on RRNA Sequences Coding the Internal Transcribed Spacers (ITS) and 5·8S Regions. Med. Mycol..

[153] Tabassum S., Zia M., Khoja A.A., David J., Iqbal M., Junaid M. (2021). Lepra Reactions: A Study of 130 Cases from Pakistan. J. Pak. Med. Assoc..

[154] Koehler A., Scroferneker M.L., Pereira B.A.S., de Souza N.M.P., Cavalcante R.D.S., Mendes R.P., Corbellini V.A. (2022). Using Infrared Spectroscopy of Serum and Chemometrics for Diagnosis of Paracoccidioidomycosis. J. Pharm. Biomed. Anal..

[155] Thompson G.R., Le T., Chindamporn A., Kauffman C.A., Alastruey-Izquierdo A., Ampel N.M., Andes D.R., Armstrong-James D., Ayanlowo O., Baddley J.W. (2021). Global Guideline for the Diagnosis and Management of the Endemic Mycoses: An Initiative of the European Confederation of Medical Mycology in Cooperation with the International Society for Human and Animal Mycology. Lancet Infect. Dis..

[156] Naranjo M.S., Trujillo M., Munera M.I., Restrepo P., Gomez I., Restrepo A. (1990). Treatment of Paracoccidioidomycosis with Itraconazole. Med. Mycol..

[157] Caceres D.H., Echeverri Tirado L.C., Bonifaz A., Adenis A., Gomez B.L., Flores C.L.B., Canteros C.E., Santos D.W., Arathoon E., Soto E.R. (2022). Current Situation of Endemic Mycosis in the Americas and the Caribbean: Proceedings of the First International Meeting on Endemic Mycoses of the Americas (IMEMA). Mycoses.

[158] Domínguez-Gil Hurlé A., Sánchez Navarro A., García Sánchez M.J. (2006). Therapeutic Drug Monitoring of Itraconazole and the Relevance of Pharmacokinetic Interactions. Clin. Microbiol. Infect..

[159] Rauseo A.M., Mazi P., Lewis P., Burnett B., Mudge S., Spec A. (2021). Bioavailability of Single-Dose SUBA-Itraconazole Compared to Conventional Itraconazole under Fasted and Fed Conditions. Antimicrob. Agents Chemother..

[160] Pappas P.G., Spec A., Miceli M., McGwin G., McMullen R., Thompson G.R.R. (2021). An Open-Label Comparative Trial of SUBA-Itraconazole (SUBA) versus Conventional Itraconazole (c-Itra) for Treatment of Proven and Probable Endemic Mycoses (MSG-15): A Pharmacokinetic (PK) and Adverse Event (AE) Analysis. Open Forum Infect. Dis..

[161] Cavalcante R.D.S., Sylvestre T.F., Levorato A.D., de Carvalho L.R., Mendes R.P. (2014). Comparison between Itraconazole and Cotrimoxazole in the Treatment of Paracoccidiodomycosis. PLoS Negl. Trop. Dis..

[162] Borges S.R.C., da Silva G.M.S., da Costa Chambela M., Oliveira R.D.V.D., Costa R.L.B., Wanke B., do Valle A.C.F. (2014). Itraconazole vs. Trimethoprim–Sulfamethoxazole: A Comparative Cohort Study of 200 Patients with Paracoccidioidomycosis. Med. Mycol..

[163] Queiroz-Telles F., Goldani L.Z., Schlamm H.T., Goodrich J.M., Espinel-Ingroff A., Shikanai-Yasuda M.A. (2007). An Open-Label Comparative Pilot Study of Oral Voriconazole and Itraconazole for Long-Term Treatment of Paracoccidioidomycosis. Clin. Infect. Dis..

[164] Thompson G.R., Rendon A., Ribeiro dos Santos R., Queiroz-Telles F., Ostrosky-Zeichner L., Azie N., Maher R., Lee M., Kovanda L., Engelhardt M. (2016). Isavuconazole Treatment of Cryptococcosis and Dimorphic Mycoses. Clin. Infect. Dis..

[165] Seki Kioshima E., Mendonça P.D.S.B.D., de Melo Teixeira M., Grenier Capoci I.R., Amaral A., Vilugron Rodrigues-Vendramini F.A., Lauton Simões B., Rodrigues Abadio A.K., Fernandes Matos L., Soares Felipe M.S. (2021). One Century of Study: What We Learned about *Paracoccidioides* and How This Pathogen Contributed to Advances in Antifungal Therapy. J. Fungi.

[166] Sylvestre T.F., Franciscone Silva L.R., Cavalcante R.D.S., Moris D.V., Venturini J., Vicentini A.P., de Carvalho L.R., Mendes R.P. (2014). Prevalence and Serological Diagnosis of Relapse in Paracoccidioidomycosis Patients. PLoS Negl. Trop. Dis..

[167] Martins R., Marques S., Alves M., Fecchio D., Franco M.F. (1997). de Serological Follow-up of Patients with Paracoccidioidomycosis Treated with Itraconazole Using Dot-Blot, ELISA and Western-Blot. Rev. Inst. Med. Trop. Sao Paulo.

[168] Dias L.D.S., Silva L.B.R., Nosanchuk J.D., Taborda C.P. (2021). Neutrophil Cells Are Essential for The Efficacy of a Therapeutic Vaccine against Paracoccidioidomycosis. J. Fungi.

[169] Muñoz J.E., Luft V.D., Amorim J., Magalhães A., Thomaz L., Nosanchuk J.D., Travassos L.R., Taborda C.P. (2014). Immunization with P10 Peptide Increases Specific Immunity and Protects Immunosuppressed BALB/c Mice Infected with Virulent Yeasts of *Paracoccidioides brasiliensis*. Mycopathologia.

[170] Magalhães A., Ferreira K.S., Almeida S.R., Nosanchuk J.D., Travassos L.R., Taborda C.P. (2012). Prophylactic and Therapeutic Vaccination Using Dendritic Cells Primed with Peptide 10 Derived from the 43-Kilodalton Glycoprotein of *Paracoccidioides brasiliensis*. Clin. Vaccine Immunol..

[171] Taborda C.P., Urán M.E., Nosanchuk J.D., Travassos L.R. (2015). Paracoccidioidomycosis: Challenges in the Development of a Vaccine Against an Endemic Mycosis in the Americas. Rev. Inst. Med. Trop. Sao Paulo.

[172] Morais E.A., Martins E.M.D.N., Boelone J.N., Gomes D.A., Goes A.M. (2015). Immunization with Recombinant Pb27 Protein Reduces the Levels of Pulmonary Fibrosis Caused by the Inflammatory Response Against *Paracoccidioides brasiliensis*. Mycopathologia.

[173] González Á. (2020). The Therapy of Pulmonary Fibrosis in Paracoccidioidomycosis: What Are the New Experimental Approaches?. J. Fungi.

[174] Finato A.C., Almeida D.F., dos Santos A.R., Nascimento D.C., Cavalcante R.S., Mendes R.P., Soares C.T., Paniago A.M.M., Venturini J. (2020). Evaluation of Antifibrotic and Antifungal Combined Therapies in Experimental Pulmonary Paracoccidioidomycosis. Med. Mycol..

[175] Puerta-Arias J.D., Pino-Tamayo P.A., Arango J.C., Salazar-Peláez L.M., González A. (2018). Itraconazole in Combination with Neutrophil Depletion Reduces the Expression of Genes Related to Pulmonary Fibrosis in an Experimental Model of Paracoccidioidomycosis. Med. Mycol..

[176] Puerta-Arias J.D., Pino-Tamayo P.A., Arango J.C., González Á. (2016). Depletion of Neutrophils Promotes the Resolution of Pulmonary Inflammation and Fibrosis in Mice Infected with *Paracoccidioides brasiliensis*. PLoS ONE.

[177] Boniche C., Rossi S.A., Kischkel B., Barbalho F.V., Moura Á.N.D., Nosanchuk J.D., Travassos L.R., Taborda C.P. (2020). Immunotherapy against Systemic Fungal Infections Based on Monoclonal Antibodies. J. Fungi.

[178] Boniche-Alfaro C., Kischkel B., Thomaz L., Carvalho-Gomes M.M., Lopes-Bezerra L.M., Nosanchuk J.D., Taborda C.P. (2021). Antibody- Based Immunotherapy Combined With Antimycotic Drug TMP- SMX to Treat Infection With *Paracoccidioides brasiliensis*. Front. Immunol..

